# The relationship between lipoproteins and the risk of esophageal cancer: a Mendelian randomization study

**DOI:** 10.3389/fnut.2024.1432289

**Published:** 2024-08-23

**Authors:** Jiale Cui, Rong Zhang, Lei Li

**Affiliations:** ^1^School of Basic Medical of Sciences, Shanxi Medical University, Taiyuan, Shanxi, China; ^2^The Gynecology and Obstetrics Department, Shanxi Provincial People’s Hospital, Taiyuan, Shanxi, China; ^3^The Radiotherapy Department, Shanxi Provincial People’s Hospital, Taiyuan, Shanxi, China

**Keywords:** esophageal cancer, low density lipoprotein, high density lipoprotein, triglyceride, Mendelian randomization, BMI

## Abstract

**Backgrounds and aims:**

Esophageal cancer (EC) causes approximately 508,000 deaths annually, making it a significant cause of cancer-related mortality. While previous studies have suggested an association between lipoprotein levels and EC risk, the causal relationship remains unexplored. This study aims to investigate the causal link between lipoproteins and EC using Mendelian randomization (MR).

**Methods and findings:**

This study employed MR to determine the causal effect between lipoproteins and EC risk, with body mass index (BMI) used as a confounder in multivariable MR (MVMR) analysis. Sensitivity analyses were conducted to assess the reliability of the results. Univariable MR (UVMR) analysis indicated that low-density lipoprotein (LDL) had a significant inverse association with EC risk (*p* = 0.03; OR = 0.89; 95%CI, 0.73–0.98), while high-density lipoprotein (HDL) and triglycerides showed no significant association. In the synthesis of findings across diverse datasets, LDL maintained a notable inverse association with the likelihood of EC (*p* < 0.001; OR = 0.89; 95%CI, 0.84–0.94). Triglyceride levels indicated a potential trend toward an adverse correlation with EC susceptibility (*p* = 0.03; OR = −0.94; 95%CI, 0.89–0.99), whereas HDL levels did not establish a definitive causal link with the occurrence of EC. MVMR analysis, adjusting for BMI, confirmed these findings.

**Conclusion:**

LDL exhibits a clear inverse causal relationship with EC risk, regardless of BMI adjustment. No causal effects were observed for HDL in relation to EC risk. Meanwhile, there is a small but statistically significant causal relationship between triglycerides and EC risk.

## 1 Introduction

Globally, esophageal cancer (EC) is a major cause of cancer mortality, resulting in an estimated 508,000 deaths annually (1). Identifying the risk factors for EC is crucial for developing preventive measures. Aberrations in lipid and lipoprotein metabolism are associated with various diseases, with elevated low-density lipoprotein (LDL) levels recognized as a significant risk factor for cardiovascular disease (CVD) (2, 3). Research has indicated a strong association between lipid metabolism and cancer development, with previous studies suggesting a negative relationship between total cholesterol, LDL, and EC risk in individuals without a family history (4–9). However, these studies have not explored the causal effects of lipoproteins on EC risk.

This study utilizes the Mendelian randomization (MR) approach to investigate the potential causal relationship between lipoproteins and EC risk. We categorized lipoproteins into high-density lipoprotein (HDL), LDL, and triglycerides, conducting separate MR analyses for each category. Considering the significant role of obesity in both lipoprotein levels and EC risk (10), we incorporated body mass index (BMI) as a confounder.

The MR approach employs genetic predictors as instrumental variables (IVs) to explore causal relationships between diseases and risk factors (11). The random allocation of genetic variants at conception serves as a surrogate for randomized controlled trials, helping to avoid biases inherent in conventional observational studies (12). Using publicly available data, we selected MR as our primary analytical method to explicitly evaluate the causal links between lipoproteins and EC risk.

## 2 Materials and methods

### 2.1 Genetic variants associated with lipoproteins

In this research, lipoproteins were categorized into HDL, LDL, and triglycerides for analysis. Single-nucleotide polymorphisms (SNPs) for these lipoproteins were obtained from the Global Lipids Genetics Consortium, primarily compiled by Willer CJ, Schmidt EM, and Sengupta S, and published in Nature Genetics (13) which published in 2013. This dataset included over 2.4 million SNPs from 188,578 samples. Simultaneously, we incorporated Global Lipids Genetics Consortium’s most recent SNPs data from the year 2023 into the analysis to validate our conclusion (14). SNPs related to body mass index (BMI) were sourced from a meta-analysis published in Human Molecular Genetics, conducted by Pulit SL in 2019 (15). This dataset contained 694,648 samples and more than 27.4 million SNPs.

For further analysis and confirm our conclusion, we included 14 datasets from the IEU database,^1^ specifically:

- HDL: ieu-a-299, ieu-a-780, ieu-b-109, ieu-b-4843, ieu-b-4844, met-d-HDL_C, ukb-e-30780.
- LDL: ieu-a-300, ieu-a-781, ieu-b-110, ieu-b-4846, ukb-e-30780.
- Triglycerides: ieu-a-302, ieu-a-783, ieu-b-111, ieu-b-4849, ieu-b-4850, met-c-934, ukb-e-30870.

Instrumental variables (IVs) were identified from all SNPs that exhibited a correlation with exposures, achieving the genome-wide association studies (GWAS) statistical significance threshold (*p* < 5 × 10^−8^) (Supplementary Tables S1–S3). A linkage disequilibrium analysis was conducted to ensure the independence of IVs, using a threshold of *r*^2^ < 0.001. Additionally, we verified that each IV had an *F*-statistic value greater than 10 (*F* > 10), confirming the reliability of the IVs. Comprehensive details regarding the sources are presented in Table 1.

**Table 1:** Details on the characteristics of each included dataset.

Phenotype	Data source	Total sample size	Population	#SNPs
Lipoprotein traits: HDL, LDL, triglyceride	Global Lipids Genetics Consortium, Willer CJ, Schmidt EM, Sengupta S, Peloso GM, Gustafsson S, et al. Discovery and refinement of loci associated with lipid levels. Nat Genet. 2013;45(11):1274–83 (13).	188,587	European	2.4 M
Esophageal cancer	Sakaue S, Kanai M, Tanigawa Y, et al. A cross-population atlas of genetic associations for 220 human phenotypes. Nat Genet. 2021;53(10):1415–24 (16).	998 cases, 475,308 controls	European	24.2 M
BMI	Pulit SL, Stoneman C, Morris AP, et al. Meta-analysis of genome-wide association studies for body fat distribution in 694,649 individuals of European ancestry. Hum Mol Genet. 2019;28(1):166–74 (15).	694,648	European	27.4 M

### 2.2 GWAS summary data on EC

The dataset for esophageal cancer (EC) was sourced from an article published in Nature Genetics in 2021, with the main analysis conducted by Sakaue (16). This dataset comprised 476,306 samples and included 24.2 million SNPs (Table 1).

### 2.3 Statistical analyses

**Figure 1:**
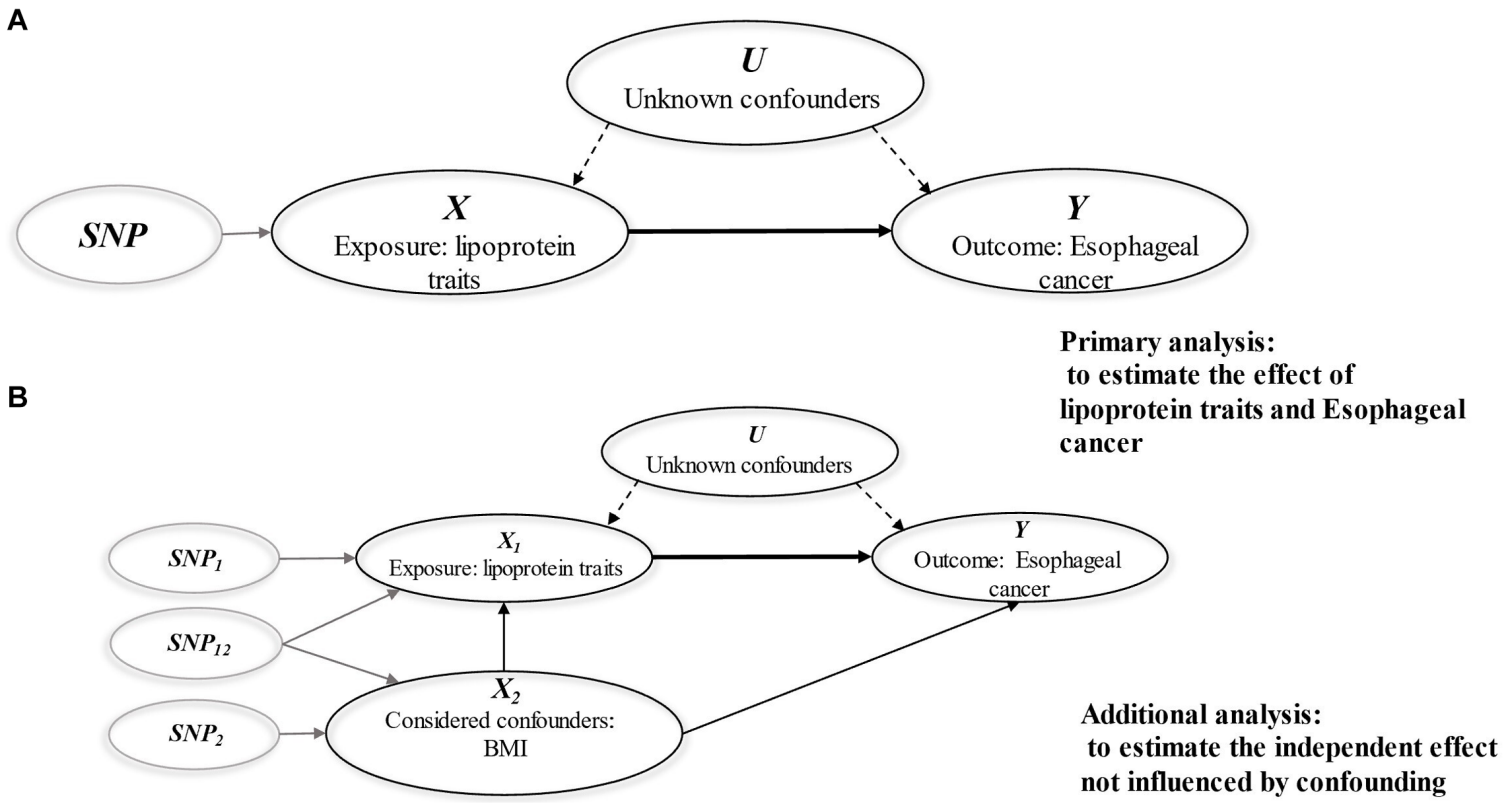
Diagrams illustrating associations examined in this study. **(A)** Univariable MR analysis; **(B)** Multivariable MR analysis. The assumptions of MR analysis: (i) The genetic variant is associated with the exposure in question (correlation assumption). (ii) The genetic variant influences the outcome solely through its effect on the exposure, with no direct effect on the outcome (the exclusion restriction assumption). (iii) The genetic variant is not correlated with other confounding factors that influence the outcome (the independence assumption).

Two-sample Mendelian randomization (MR) analysis was utilized to explore the potential causal effect between lipoproteins and the risk of esophageal cancer (EC). MR studies use genetic variability as an instrumental variable (IV) and must meet specific criteria for IV assumptions. These criteria are:

- The genetic variant is associated with the exposure in question (correlation assumption).
- The genetic variant influences the outcome solely through its effect on the exposure, with no direct effect on the outcome (the exclusion restriction assumption).
- The genetic variant is not correlated with other confounding factors that influence the outcome (the independence assumption) (Figure 1) (17).

In our Mendelian randomization (MR) analyses, we primarily employed the inverse-variance weighted (IVW) method. Additionally, the weighted median (WM) model and MR-Egger regression were utilized to validate the consistency of the causal estimates. The IVW approach treats each SNP as an independent natural experiment influencing the outcome and combines these individual effects, using the results as weights to determine the overall causal effect. The fixed-effect model IVW analysis provides an unbiased assessment in the absence of horizontal pleiotropy or if horizontal pleiotropy is balanced (18). In scenarios with heterogeneity, we applied the multiplicative random-effects IVW model, which yields a valid estimate assuming balanced pleiotropy (19, 20).

For pleiotropy assessment, we used the MR-Egger intercept test, and to gauge heterogeneity among genetic variants, we applied Cochran’s *Q* statistic (21). Additionally, leave-one-out analyses were conducted to ensure our results were not driven by a single SNP.

Given the substantial influence of obesity on lipoprotein levels and EC risk, we performed a multivariable Mendelian randomization (MVMR) analysis to evaluate the causal effect of lipoproteins on EC risk while adjusting for BMI. The MVMR extension of the IVW method was used to correct for pleiotropy (22).

The results were presented as odds ratios (ORs) with corresponding 95% confidence intervals (CIs). Meanwhile, in the meta-analysis, the results were presented in the form of beta values [*β* = log (OR)]. All analyses were conducted using R (version 4.2.2) and the “TwoSampleMR” package.

## 3 Results

### 3.1 GWAS analysis

**Figure 2:**
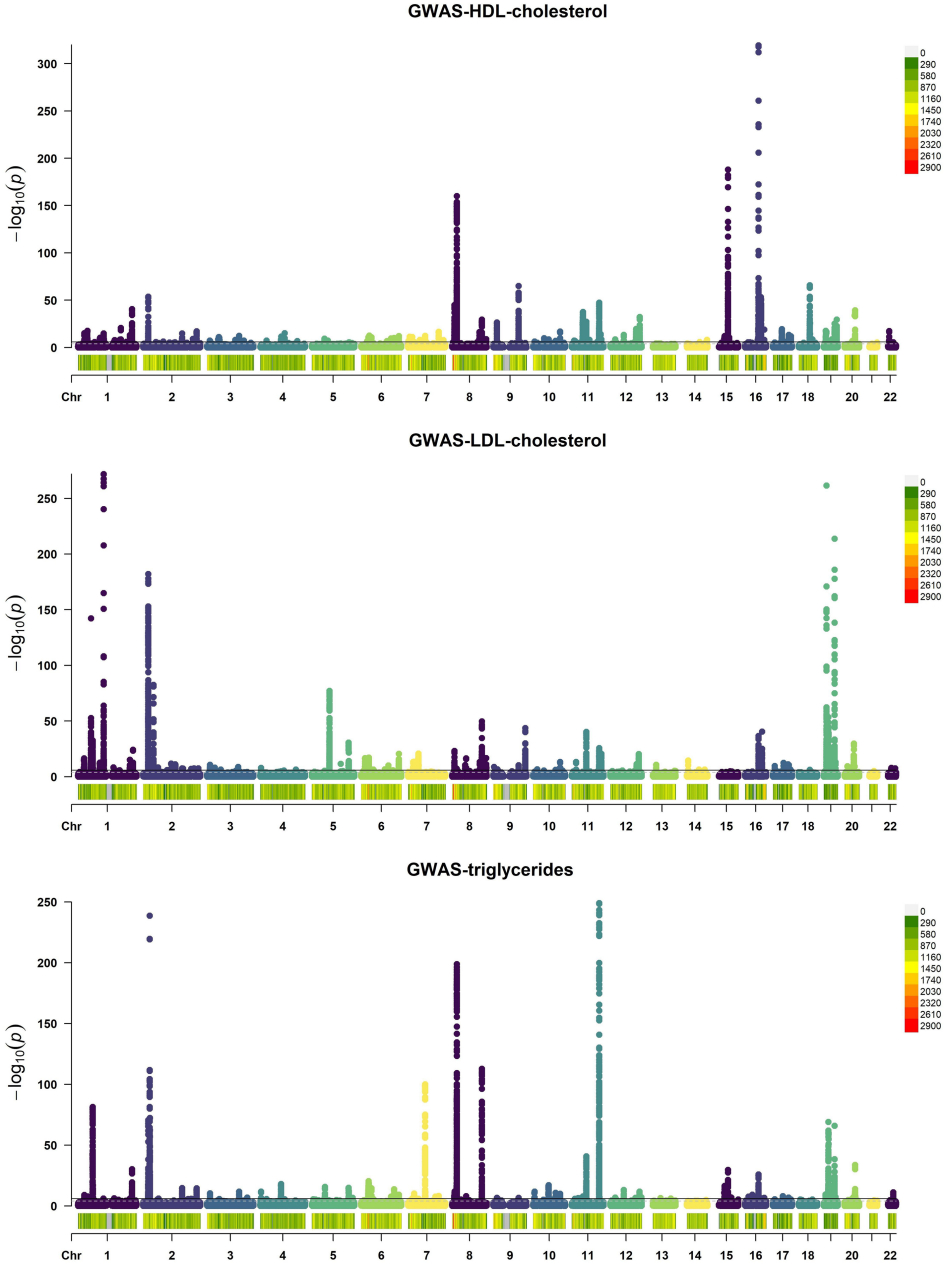
Manhattan Plots of GWAS results of HDL, LDL, and triglycerides. Each point on the graph denotes a single SNP, mapping its chromosome location along the *x*-axis and its association with HDL, LDL, and triglycerides via the −log10 *p*-value on the *y*-axis. A black horizontal line indicates the genome-wide significance threshold at *p* = 5 × 10^−8^.

Upon examining the GWAS data, we identified 116 SNPs in HDL, 93 in LDL, and 70 in triglycerides that surpassed the genome-wide significance level of *p* < 5 × 10^−8^, as indicated by the Manhattan Plots (Figure 2). These SNPs, meeting the stringent threshold, are considered genomically significant and serve as potential instrumental variables for further study.

**Figure 3:**
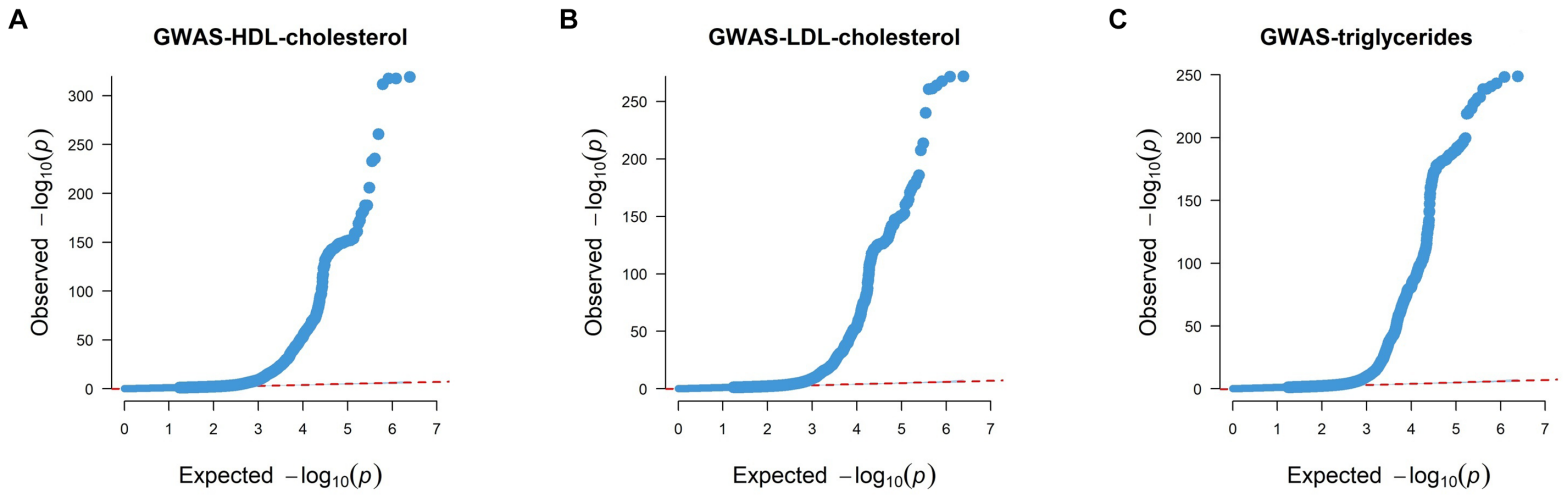
Quantile–Quantile plots of GWAS results of HDL, LDL, and triglycerides. **(A)** QQ plot of HDL’s GWAS dataset. **(B)** QQ plot of LDL’s GWAS dataset. **(C)** QQ plot of triglycerides’ GWAS dataset. Each point on the graph denotes a single SNP, the *x*-axis in the figure represents the expected *p* value, and the y-axis represents the observed *p* value, both of which are represented by the *p*-value −log10. The red dashed line roughly illustrates the pattern of random genetic drift across the genome.

We also generated QQ (Quantile–Quantile) plots using GWAS data, with the findings detailed below. The red dashed line illustrates the pattern of random genetic drift across the genome. For HDL, LDL, and triglycerides, the QQ plots showed a quick divergence from observed to expected values once the expected −log10P exceeded 3 (Figure 3). This suggests that the SNPs examined are not a result of genetic drift and that a significant correlation exists between phenotype and genotype due to natural selection. This confirms the suitability of the GWAS data for our analysis.

### 3.2 Univariable Mendelian randomization analysis

All *F*-statistics for the SNPs used in our analysis were significantly greater than 10, indicating robust instrumental variables. The IVW method revealed that for each standard deviation increase in LDL level, there was a 15% reduction in the risk of EC (OR = 0.85; 95%CI, 0.73–0.98; *p* = 0.03). The WM approach yielded results consistent with those from the IVW method (Table 2). The MR-Egger approach also indicated a strong negative association between LDL and the risk of EC (OR = 0.91; 95%CI, 0.71–1.16).

**Table 2:** Causal effects of lipoprotein traits on esophageal cancer.

Exposure	HDL cholesterol		LDL cholesterol		Triglycerides	
IVW method (fixed effects)	OR (95%CI)	1.12 (0.95–1.31)	OR (95%CI)	0.85 (0.73–0.98)	OR (95%CI)	0.86 (0.72–1.02)
	*p*-value	0.2	*p*-value	0.03	*p*-value	0.08
MR Egger	OR (95%CI)	0.95 (0.69–1.30)	OR (95%CI)	0.91 (0.71–1.16)	OR (95%CI)	0.97 (0.73–1.28)
	*p*-value	0.7	*p*-value	0.4	*p*-value	0.8
Weighted median	OR (95%CI)	1.03 (0.79–1.33)	OR (95%CI)	0.73 (0.58–0.92)	OR (95%CI)	1.07 (0.82–1.41)
	*p*-value	0.8	*p*-value	0.009	*p*-value	0.6

However, neither the IVW nor the WM method showed a statistically significant causal effect between HDL and the risk of EC (IVW: *p* = 0.2, OR = 1.16; 95%CI, 0.95–1.31; WM: *p* = 0.8, OR = 1.03; 95%CI, 0.97–1.33). Similar findings were observed for triglycerides in the IVW and WM analyses (IVW: *p* = 0.08, OR = 0.86; 95%CI, 0.72–1.02; WM: *p* = 0.6, OR = 1.07; 95%CI, 0.82–1.41).

These findings (Table 2) suggest a causal relationship between LDL and EC risk, while indicating no causal effect for HDL or triglycerides on EC risk.

### 3.3 Sensitivity analyses

#### 3.3.1 Sensitivity analyses of lipoproteins

To assess heterogeneity, we employed Cochran’s *Q* test. For investigating horizontal pleiotropy, we used the MR-Egger regression approach, which revealed no evidence of directional pleiotropy (Table 3). Cochran’s *Q* test for LDL exhibited statistical significance (*p* = 0.1). As a result, we applied a multiplicative random-effects model to reassess the Mendelian randomization effect of LDL. This analysis confirmed a causal relationship and identified a direct negative correlation between LDL levels and esophageal cancer risk (*p* = 0.04, OR = 0.85; 95%CI, 0.73–0.98).

**Table 3:** Sensitivity analyses.

Exposure	HDL cholesterol		LDL cholesterol		Triglycerides	
IVW method (multiplicative random effects)	OR (95%CI)	1.12 (0.95–1.31)	OR (95%CI)	0.85 (0.73–0.99)	OR (95%CI)	0.86 (0.72–1.03)
	*p*-value	0.2	*p*-value	0.04	*p*-value	0.1
MR-Egger regression analysis	Intercept	0.8 × 10^−2^	Intercept	−0.5 × 10^−2^	Intercept	−0.8 × 10^−2^
	*p*-value	0.2	*p*-value	0.5	*p*-value	0.3
Cochran’s *Q* test	*Q* statistic	119	*Q* statistic	101	*Q* statistic	75
	*p*-value	0.3	*p*-value	0.1	*p*-value	0.2

#### 3.3.2 Leave-one-out analysis

The Leave-One-Out analysis showed that the risk assessments for HDL, LDL, and triglycerides related to esophageal cancer remained consistent when each SNP was removed sequentially (see Supplementary Tables S4–S6).

### 3.4 Mendelian randomization-meta analysis

**Figure 4:**
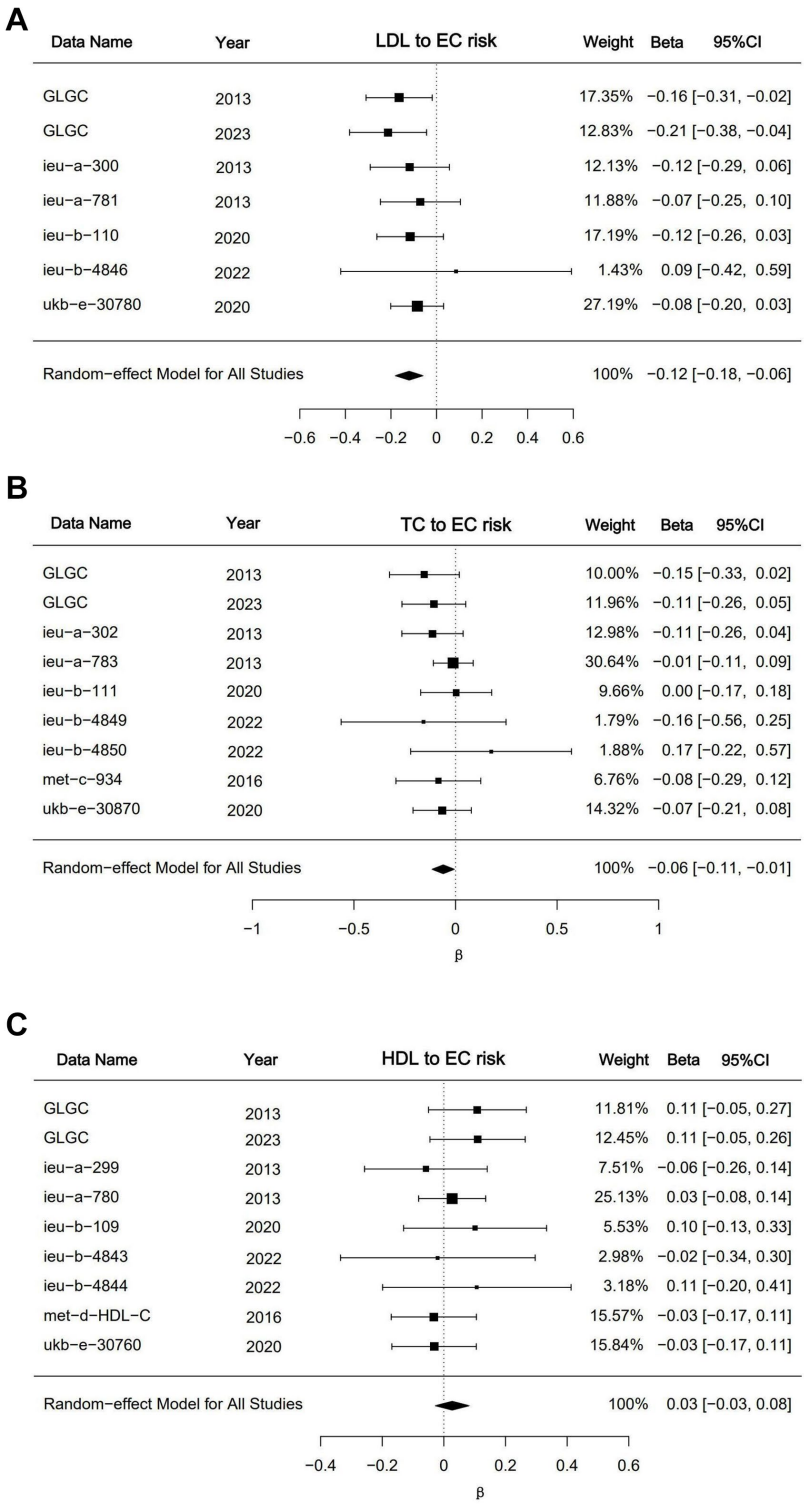
Meta analysis results for LDL, HDL and triglycerides. **(A)** Forest plot of meta result for LDL datasets; **(B)** forest plot of meta result for triglyceride datasets; **(C)** forest plot of meta result for triglyceride datasets. The “DATA” shows the dataset’s GWAS ID in the IEU database or data sources. The GLGC means Global Lipids Genetics Consortium. The results were presented as *β* with corresponding 95%CIs and *p*-values.

To substantiate our findings and mitigate potential biases arising from reliance on a single dataset, we performed a meta-analysis incorporating the IVW outcomes of multiple dataset analyses (Figure 4). The meta-analytic outcomes for LDL and HDL align with the conclusions derived from univariate Mendelian randomization, indicating that LDL acts as a protective factor against EC (*p* < 0.001; *β* = −0.12; 95%CI, −0.18, −0.06), whereas no significant causal relationship was observed between HDL and EC (*p* = 0.3; *β* = −0.12; 95%CI, −0.03, 0.08). In the meta-analysis of triglyceride data, while individual datasets did not yield statistically significant findings, the pooled analysis revealed a modest yet statistically significant inverse causal relationship between triglyceride levels and the incidence of EC (*p* = 0.03; *β* = −0.06; 95%CI, −0.11, −0.01). The outcomes of the analyses conducted on these datasets have withstood sensitivity assessments, thereby affirming the robustness of the derived conclusions. The comprehensive details of the remaining meta-analysis results will be included in the supplementary documentation accompanying the main text.

### 3.5 Multivariable Mendelian randomization analysis

Given the significant impact of BMI on lipoprotein levels and esophageal cancer risk, we performed a Multivariable Mendelian Randomization (MVMR) analysis. This method allowed us to estimate the causal effect of LDL on esophageal cancer risk while adjusting for BMI.

**Figure 5:**
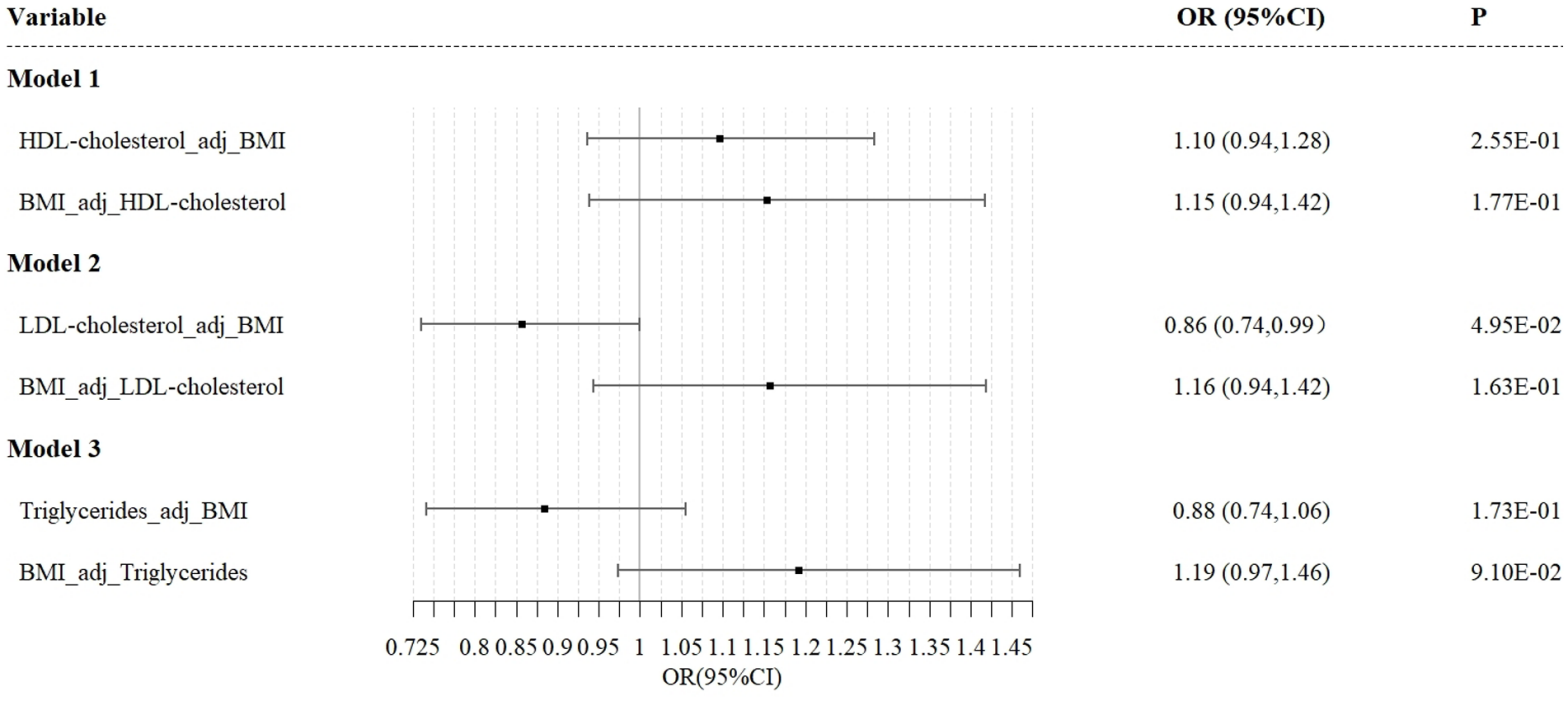
Independent effect of lipoprotein traits on the risk of esophageal cancer using multivariable Mendelian randomization analysis. Model 1: independent effect of HDL cholesterol on esophageal cancer after adjusting BMI; Model 2: independent effect of LDL cholesterol on esophageal cancer after adjusting BMI; Model 3: independent effect of triglycerides on esophageal cancer after adjusting BMI.

The results from the MVMR analysis using the IVW method demonstrate that LDL maintains a direct causal association with esophageal cancer risk when BMI is accounted for (OR = 0.74; 95%CI, 0.64–0.89; *p* = 0.05). Conversely, the MVMR analyses for HDL and triglycerides did not indicate a direct causal effect on esophageal cancer risk (Figure 5).

## 4 Discussion

This study utilizes Mendelian randomization to elucidate the causal associations between lipoproteins and esophageal cancer (EC) risk. Our UVMR analysis reveals a significant causal relationship between LDL levels and EC risk, characterized by a negative association. In contrast, HDL and triglycerides did not demonstrate a causal effect on EC risk. However, in subsequent meta-analyses incorporating multiple datasets, triglyceride levels demonstrated a statistically significant causal impact on the incidence of EC. Thus, our findings suggest that LDL and triglycerides may serve as a protective factor against EC, while HDL do not show a direct causal impact on EC risk.

The relationship between lipoproteins and cancer risk has been actively researched. Previous studies employing traditional statistical methods have identified a significant negative correlation between LDL levels and EC risk, particularly among individuals with a family history of the disease (23). However, confounding factors such as BMI, alcohol consumption, or smoking may affect the observed relationship between LDL and EC risk, potentially leading to biased interpretations.

Our conclusion is supported by existing research, including reports by Emberson and Muntoni, which indicate that cancer patients often have reduced LDL levels (24, 25). Additionally, some studies have linked low LDL levels to an increased risk and mortality from neoplasms (26–29). However, these findings contrast with a previous study suggesting that LDL’s protective effect against esophageal cancer was observed only in individuals without a family history of the disease (23). Differences in findings may stem from variations in statistical methodologies, as randomized controlled trials (RCTs) might inadvertently include confounding variables in their results. We hypothesize that genetic factors specific to individuals with a family history of cancer could influence the causal relationship between lipoproteins and esophageal cancer, potentially leading to biased outcomes. Since a family history of cancer is a recognized proxy for genetic susceptibility (30), this hypothesis seems plausible.

Our analysis found significant reverse association between triglycerides and esophageal cancer risk. This is consistent with prior research reporting a significant inverse relationship between triglyceride levels and cancer risk (31). Meanwhile, Tomiki’s research suggests that low triglyceride levels may be a consequence of low LDL (32). This implies that any apparent negative correlation between triglycerides and esophageal cancer may be mediated by LDL, which may could explain why triglycerides can reduce the risk of EC. In the analysis of UVMR, triglycerides did not exhibit a protective effect on the incidence of EC risk. However, upon comprehensive analysis of multiple datasets spanning various years and ethnicities, and amalgamating the respective effect estimates, a statistically significant causal relationship between triglyceride levels and EC integrity was discerned. This finding underscores the potential for biased outcomes in studies with insufficient sample sizes within a single triglyceride dataset. Conversely, augmenting the sample size for analysis enhances the robustness and credibility of the conclusions. Collectively, these findings suggest that triglycerides may indeed serve as a protective factor against EC risk. Furthermore, our findings align with previous research indicating no significant relationship between HDL and esophageal cancer risk (31).

The existing literature employing Mendelian randomization to investigate the nexus between lipoproteins and EC did not reveal a significant causal association between the three lipoproteins and EC, presenting a marked discrepancy with our findings (33). We conjecture that this discrepancy may be attributed to the data types utilized. The prior research utilized individual-level data from UK Biobank and Biobank Japan as exposures, whereas our study employed summary-level data. The variation in data types might have contributed to the divergent research outcomes. Furthermore, the previous study sampled populations from Asia and Europe, and demographic disparities may have also introduced bias into the results.

Previous meta-analyses have identified a J-shaped relationship between BMI and esophageal cancer (EC) risk (10). This particular study included approximately 1 million participants from 44 cohorts. Data were collected through questionnaires, anthropometric assessments, and laboratory tests, covering risk factors, medical histories, and family backgrounds. The study also included follow-up assessments for newly diagnosed cancer cases and mortality rates. The analysis found increased mortality from esophageal cancer among participants with a BMI below the normal range (18.5–23 kg/m^2^) and those in the extremely obese category (BMI > 35 kg/m^2^), supporting a J-shaped relationship between BMI and EC-related mortality. Additionally, a significant correlation was observed between BMI and lipoprotein levels, with obesity associated with elevated LDL, HDL, and triglycerides (34). Given these findings, BMI was included as a covariate in our MVMR analysis. The results confirmed that the causal association between LDL levels and EC risk remained significant even after adjusting for BMI, thereby reinforcing the validity of our conclusions.

Regarding the potential protective effect of LDL on esophageal cancer (EC) risk, this study hypothesizes that it may be related to the role of oxidized LDL (ox-LDL). Prior research suggests that ox-LDL can cause DNA damage and disrupt repair mechanisms, leading to significant DNA degradation (35). Additionally, ox-LDL affects intracellular redox balance by influencing key pathways (36) and increases the activity of Protein Kinase C (PKC), which enhances the function of p21-ras proteins, contributing to tumor development (37).

We propose that higher levels of LDL may mitigate the effects of ox-LDL by reducing its concentration in the plasma, thereby inhibiting cancer progression. Furthermore, we speculate that the protective effect of LDL on EC risk could be mediated through the scavenger receptor lectin-like oxidized low-density lipoprotein receptor-1 (LOX-1). LOX-1 has been shown to initiate autophagy processes that significantly contribute to EC development (38). As a primary scavenger receptor, LOX-1 binds to ox-LDL, which can upregulate LOX-1 expression (39). We suggest that increased LDL levels might reduce ox-LDL levels, leading to decreased LOX-1 expression and thus providing a protective effect against EC.

Some studies have proposed that LDL may contribute to cancer risk by supplying lipids and cholesterol that fuel cancer cell growth (40). However, our research posits that LDL’s role in cancer risk elevation as a nutrient source for tumor cells is not substantial. We argue that LDL’s impact on inhibiting tumor cell proliferation by reducing ox-LDL or LOX-1 levels and enhancing specific molecular pathways significantly outweighs its role as a nutrient for cancer cells.

The findings of this comprehensive investigation suggest that LDL may exert a protective role against the onset of esophageal cancer. This novel insight could potentially position LDL as an innovative prognostic indicator for esophageal cancer in clinical contexts. The presence of reduced levels of LDL proteins may serve as a significant biomarker, indicative of an increased susceptibility to esophageal cancer. Recognition of this association could prove instrumental in enabling healthcare professionals and patients to adopt earlier and more comprehensive preventive strategies, thereby enhancing the overall management and prognosis of this disease.

In clinical practice, LDL is commonly recognized as a primary pathogenic factor in cardiovascular diseases (41). Previous research has established LDL as a key etiological factor in several cardiovascular conditions, including atherosclerosis (42). Given the potential protective role of LDL against esophageal cancer, it is plausible to hypothesize an unexplored causal relationship between esophageal cancer and cardiovascular disease. Supporting this hypothesis, a Registry-Based Cohort Study observed a transient increase in cardiovascular disease incidence within 1 year following an esophageal cancer diagnosis (43). This observation highlights the potential clinical relevance of our hypothesis and underscores the need for further research to clarify the relationship between cardiovascular disease and esophageal cancer.

The strengths of this study lie in the application of Mendelian randomization (MR) techniques, which mitigate the influence of confounding variables on the observed association between exposure and outcome. This methodological approach enhances the robustness of our findings and allows for a more rigorous exploration of causal links between specific factors and outcomes. Additionally, incorporating BMI as a covariate in the multivariable Mendelian randomization (MVMR) analysis corroborates the causal relationship between LDL levels and esophageal cancer risk, further validating the conclusions and enhancing the reliability of our results. Simultaneously, this research integrated diverse analytical approaches and consolidated the resulting effect measures via meta-analytic procedures, aiming to corroborate or enhance our insights. Such an approach effectively mitigates the bias that can arise from the diverse years or ethnic compositions within individual dataset, thereby bolstering the robustness of the article’s conclusions.

However, this study has its limitations. First, the complex regulatory network of lipoproteins presents challenges in precisely disentangling LDL’s protective mechanisms concerning esophageal cancer. Although plausible hypotheses have been proposed, they remain speculative without experimental or analytical validation. Secondly, we cannot overcome the bias caused by collinearity in multivariate analysis, so we cannot include as many variables as possible in one analysis to make our results more reliable. To address these limitations, a multidisciplinary approach combining bioinformatics and laboratory experimentation is recommended to validate and elucidate LDL’s protective mechanisms against esophageal cancer. Concomitantly, the integration of statistical methods is anticipated to enhance the efficacy of predictive models.

Future research should focus on further investigating the intricate relationship between lipoproteins and esophageal cancer risk. Concurrently, exploring the potential link between cardiovascular disease and esophageal cancer could provide valuable insights for clinical practice and enhance our understanding of these conditions.

## Data Availability

The original contributions presented in the study are included in the article/Supplementary material, further inquiries can be directed to the corresponding author.

## Footnotes

^1^https://gwas.mrcieu.ac.uk/
