# Supplementary material for: The relationship between lipoproteins and the risk of esophageal cancer: a Mendelian randomization study
Source: Front Nutr. 2024 Aug 23;11:1432289. doi: 10.3389/fnut.2024.1432289 (PMC11377315; doi:10.3389/fnut.2024.1432289)
Supplement: Supplementary file 1 [file Data_Sheet_1.ZIP › SupMaterial/Detailed Result of Meta analysis/META result-ldl.docx]

Random-Effects Model (k = 7; tau^2 estimator: DL)

logLik deviance AIC BIC AICc

8.8318 2.8066 -13.6636 -13.7718 -10.6636

tau^2 (estimated amount of total heterogeneity): 0 (SE = 0.0040)

tau (square root of estimated tau^2 value): 0

I^2 (total heterogeneity / total variability): 0.00%

H^2 (total variability / sampling variability): 1.00

Test for Heterogeneity:

Q(df = 6) = 2.8066, p-val = 0.8327

Model Results:

estimate se zval pval ci.lb ci.ub

-0.1201 0.0309 -3.8914 <.0001 -0.1806 -0.0596 ***

---

Signif. codes: 0 ‘***’ 0.001 ‘**’ 0.01 ‘*’ 0.05 ‘.’ 0.1 ‘ ’ 1
