# Supplementary material for: The relationship between lipoproteins and the risk of esophageal cancer: a Mendelian randomization study
Source: Front Nutr. 2024 Aug 23;11:1432289. doi: 10.3389/fnut.2024.1432289 (PMC11377315; doi:10.3389/fnut.2024.1432289)
Supplement: Supplementary file 1 [file Data_Sheet_1.ZIP › SupMaterial/UVMR for DATA from GLGC-2023/GLGC-2023 RESULT/RESULT.docx]

| **Trait** | **Method** | **OR(95%CI)** | **P-value** |
| --- | --- | --- | --- |
| HDL | IVW(multiplicative random effects) | 1.116 (0.952-1.307) | 0.18 |
|  | MR Egger | 1.096 (0.840-1.430) | 0.5 |
|  | Weighted median | 1.075 (0.814-1.420) | 0.61 |
|  | IVW (fixed effects) | 1.116 (0.956-1.302) | 0.17 |
| LDL | IVW(multiplicative random effects) | 0.808 (0.681-0.960) | 1.50E-02 |
|  | MR Egger | 0.731 (0.547-0.976) | 3.40E-02 |
|  | Weighted median | 0.712 (0.536-0.944) | 1.80E-02 |
|  | IVW (fixed effects) | 0.808 (0.683-0.957) | 1.40E-02 |
| TC | IVW(multiplicative random effects) | 0.899 (0.764-1.058) | 0.2 |
|  | MR Egger | 0.868 (0.661-1.140) | 0.31 |
|  | Weighted median | 0.923 (0.702-1.213) | 0.56 |
|  | IVW (fixed effects) | 0.899 (0.768-1.052) | 0.18 |
