# Supplementary figures and images for: The relationship between lipoproteins and the risk of esophageal cancer: a Mendelian randomization study
Source: Front Nutr. 2024 Aug 23;11:1432289. doi: 10.3389/fnut.2024.1432289 (PMC11377315; doi:10.3389/fnut.2024.1432289)

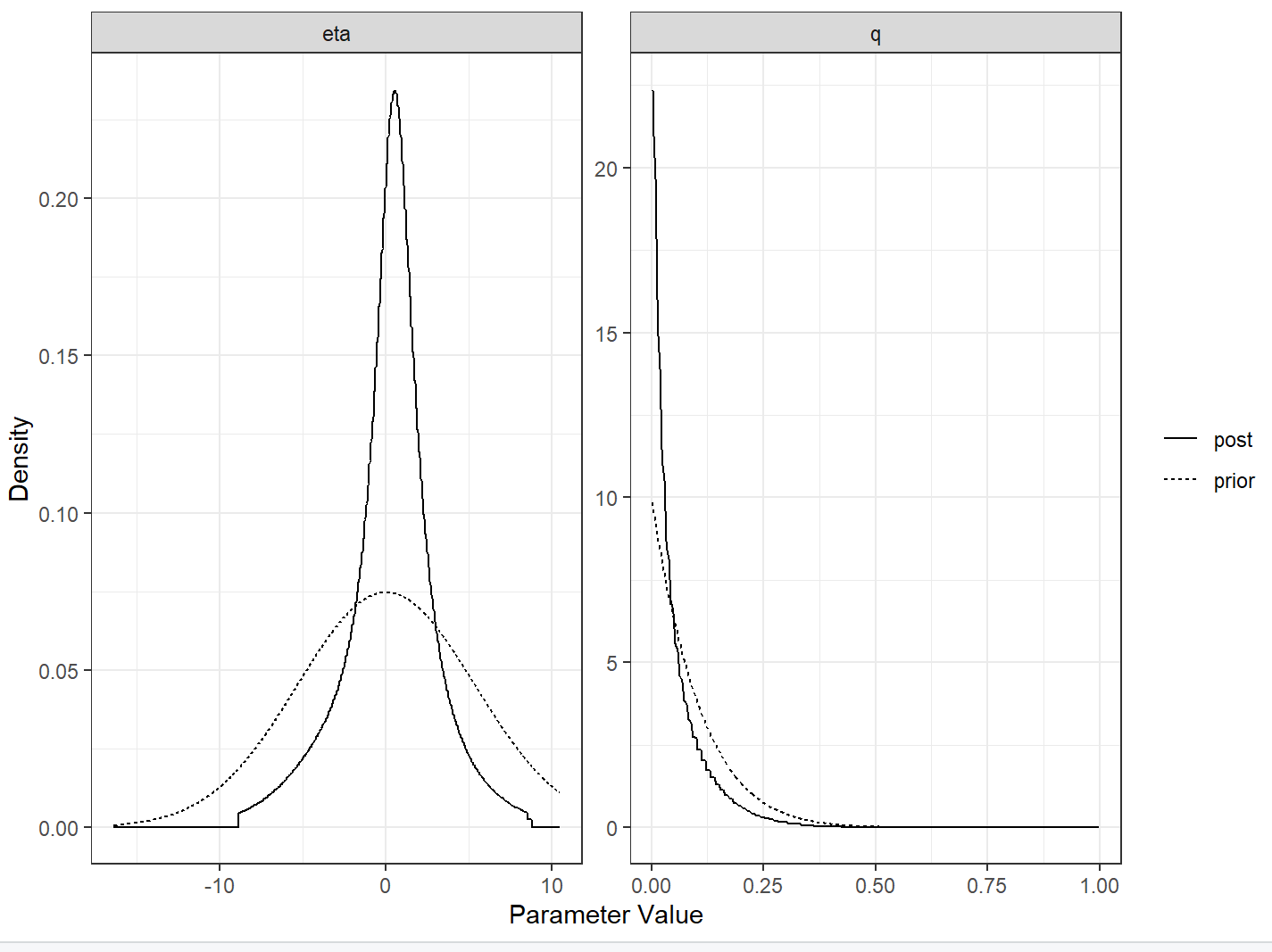

Supplement: Supplementary file 1 [file Data_Sheet_1.ZIP › SupMaterial/MR-Cause results/result 1.png]

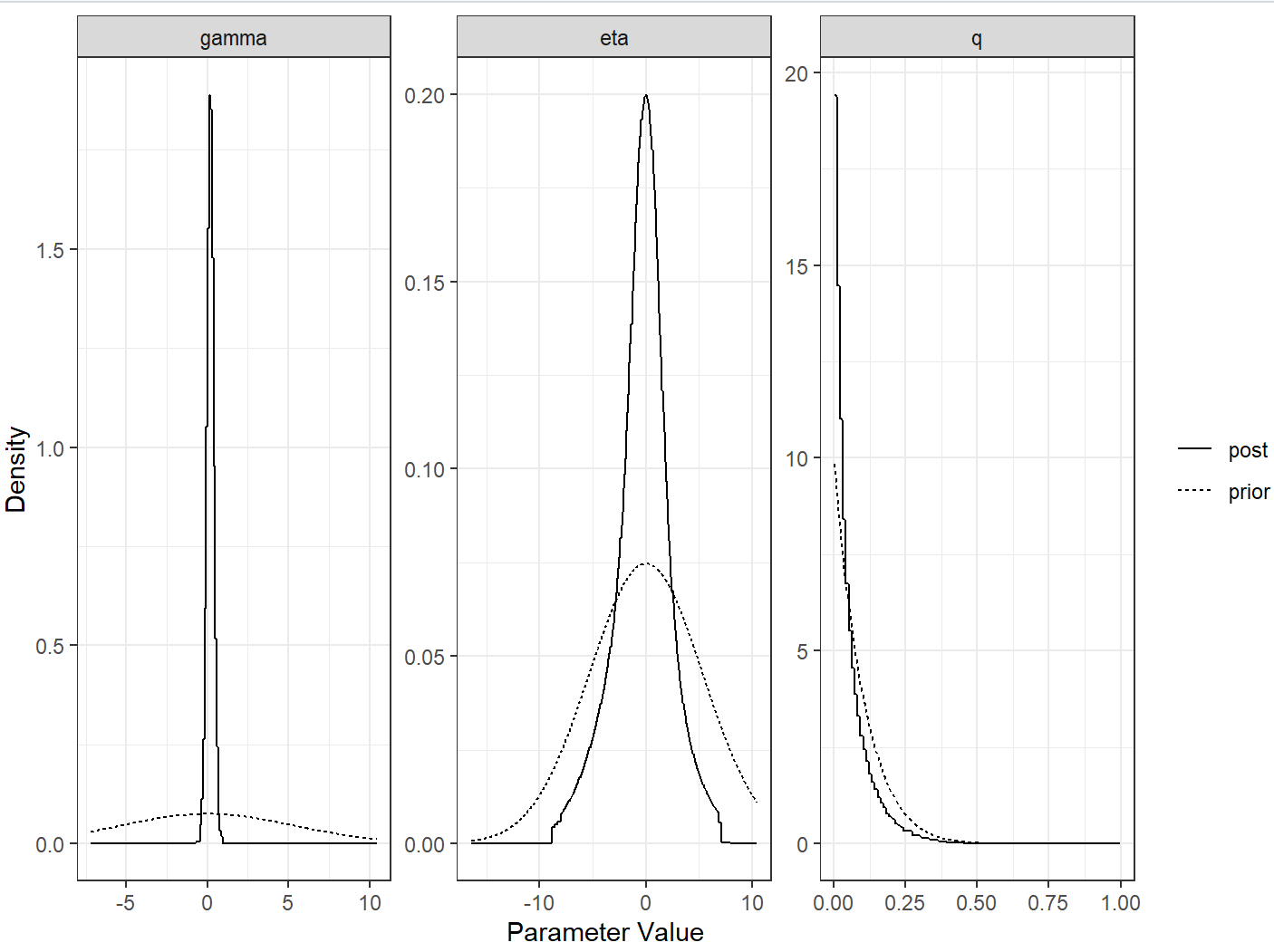

Supplement: Supplementary file 1 [file Data_Sheet_1.ZIP › SupMaterial/MR-Cause results/result2.png]

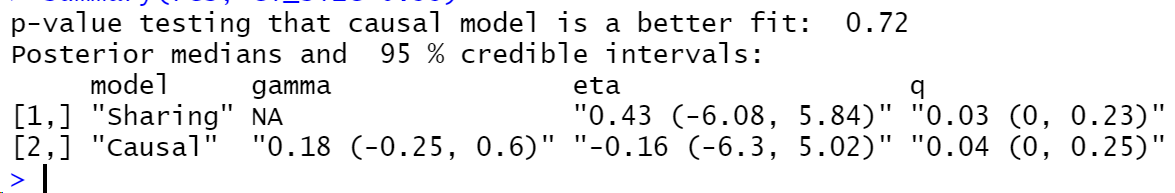

Supplement: Supplementary file 1 [file Data_Sheet_1.ZIP › SupMaterial/MR-Cause results/result3.png]
